# Mental health conditions in people affected by filarial lymphoedema in Malawi: prevalence, associated risk factors and the impact of an enhanced self-care intervention

**DOI:** 10.1093/inthealth/ihad064

**Published:** 2023-12-20

**Authors:** Carrie Barrett, John Chiphwanya, Limbikani Chaponda, Dorothy E Matipula, Joseph D Turner, Mark J Taylor, Jonathan M Read, Louise A Kelly-Hope

**Affiliations:** Centre for Neglected Tropical Diseases, Department of Tropical Disease Biology, Liverpool School of Tropical Medicine, Pembroke Place, Liverpool, L3 5QA, UK; National Lymphatic Filariasis Elimination Programme, Ministry of Health, P.O. Box 30377, Lilongwe 3, Malawi; National Lymphatic Filariasis Elimination Programme, Ministry of Health, P.O. Box 30377, Lilongwe 3, Malawi; National Lymphatic Filariasis Elimination Programme, Ministry of Health, P.O. Box 30377, Lilongwe 3, Malawi; Centre for Neglected Tropical Diseases, Department of Tropical Disease Biology, Liverpool School of Tropical Medicine, Pembroke Place, Liverpool, L3 5QA, UK; Centre for Neglected Tropical Diseases, Department of Tropical Disease Biology, Liverpool School of Tropical Medicine, Pembroke Place, Liverpool, L3 5QA, UK; Lancaster Medical School, South West Drive, Bailrigg, Lancaster, LA1 4ZP, UK; Centre for Neglected Tropical Diseases, Department of Tropical Disease Biology, Liverpool School of Tropical Medicine, Pembroke Place, Liverpool, L3 5QA, UK; Department of Livestock and One Health, Institute of Infection, Veterinary and Ecological Sciences, University of Liverpool, 146 Brownlow Hill, Liverpool, L3 5RF, UK

**Keywords:** acute filarial attacks, depression, lymphatic filariasis, mental health, morbidity management, quality of life

## Abstract

**Background:**

This study aimed to determine the key mental health indicators affecting people affected by lymphatic filariasis (LF) lymphoedema by assessing the prevalence of depressive symptoms and quality of life (QOL), identifying associated sociodemographic and clinical risk factors, and evaluating the impact of an enhanced self-care intervention for lymphoedema management.

**Methods:**

A prospective cohort study of adults with filarial lymphoedema from two regions of Malawi was conducted over six months in 2021. Depressive symptoms and QOL were assessed using Patient Health Questionnaire (PHQ-9) and LF Specific QOL Questionnaire, respectively, at baseline (pre-intervention), 3- and 6-months (postintervention). Beta regression analysis identified risk factors, and assessed the impact of the intervention.

**Results:**

Three hundred eleven affected individuals were surveyed with 23% (95% CI 18%–29%) reporting mild/moderate depressive symptoms and 31% (95% CI 26%–37%) reporting moderately low/low QOL. Higher depressive symptom scores were associated with high frequency of acute filarial attack episodes. Individuals with higher depressive symptoms (Adjusted Odds Ratios (AOR) 0.93, 95% CI 0.93–0.93) and lower QOL (AOR 0.98, 0.98–0.98) showed greatest improvement in mental health indicators over 3-months but was not sustained to the same level at 6-months.

**Conclusions:**

Sustained morbidity management and psychological support is recommended for affected persons to ensure long-term positive mental health and clinical outcomes.

**Contexte:**

Cette étude vise à déterminer les principaux indicateurs de santé mentale affectant les personnes atteintes de lymphœdème dû à la filariose lymphatique (FL) en évaluant la prévalence des symptômes dépressifs et la qualité de vie (QV), en identifiant les facteurs de risque sociodémographiques et cliniques associés, et en évaluant l'impact d'une intervention améliorée d'autosoins pour la gestion du lymphœdème.

**Méthodes:**

Une étude de cohorte prospective d'adultes atteints de lymphoedème filaire dans deux régions du Malawi a été menée pendant six mois en 2021. Les symptômes dépressifs et la qualité de vie ont été évalués à l'aide du questionnaire sur la santé des patients (PHQ-9) et du questionnaire sur la qualité de vie spécifique au lymphœdème, respectivement, au début de l'étude (avant l'intervention), et à 3 puis 6 mois après l'intervention. Une analyse de régression beta a permis d'identifier les facteurs de risque et d'évaluer l'impact de l'intervention.

**Résultats:**

Trois cent onze personnes affectées ont été interrogées, dont 23% (95% CI 18%–29%) ont déclaré des symptômes dépressifs légers/modérés et 31% (95% CI 26%–37%) ont déclaré une qualité de vie modérément faible/faible. Des scores élevés de symptômes dépressifs ont été associés à une fréquence élevée d'épisodes de crises filariennes aiguës. Les personnes présentant des symptômes dépressifs plus élevés (rapport de cotes ajusté (RCA) 0.93, IC à 95 % 0.93–0.93) et une qualité de vie plus faible (RCA 0.98, 0.98–0.98) ont montré la plus grande amélioration des indicateurs de santé mentale au cours des trois mois, mais cette amélioration ne s'est pas maintenue au même niveau au cours des six mois suivants.

**Conclusion:**

Gestion de la morbidité et soutien psychologique sont des éléments clés pour garantir une santé mentale et des résultats cliniques satisfaisants de personnes atteintes sur le long terme.

**Antecedentes:**

Este estudio tuvo como objetivo determinar los indicadores clave de salud mental que afectan a las personas afectadas por linfedema por filariasis linfática (FL) mediante la evaluación de la prevalencia de síntomas depresivos y calidad de vida (CdV), la identificación de factores de riesgo sociodemográficos y clínicos asociados, y la evaluación del impacto de una intervención de autocuidado mejorada para el manejo del linfedema.

**Métodos:**

Se realizó un estudio prospectivo de cohortes de adultos con linfedema filarial de dos regiones de Malawi durante seis meses en 2021. Los síntomas depresivos y la calidad de vida se evaluaron mediante el Cuestionario de Salud del Paciente (PHQ-9) y el Cuestionario de Calidad de Vida específico para el LF Cuestionario, respectivamente, al inicio (preintervención) y a los 3 y 6 meses (posintervención). El análisis de regresión beta identificó los factores de riesgo y evaluó el impacto de la intervención.

**Resultados:**

Se encuestó a 311 afectados, de los cuales el 23% (IC 95%, 18%–29%) presentaba síntomas depresivos leves/moderados y el 31% (IC 95%, 26%–37%) una CdV moderadamente baja/baja CdV. Las puntuaciones más altas de síntomas depresivos se asociaron con una alta frecuencia de episodios de ataques agudos de filarias. Los individuos con mayores síntomas depresivos (Odds Ratios Ajustados [ORA] 0.93; IC 95%: 0.93–0.93) y menor CdV (ORA 0.98; 0.98–0.98) mostraron la mayor mejoría en los indicadores de salud mental a los 3 meses, pero no se mantuvo al mismo nivel a los 6 meses.

**Conclusiones:**

Se recomienda el manejo sostenido de la morbilidad y el apoyo psicológico a las personas afectadas para garantizar resultados clínicos y de salud mental positivos a largo plazo.

## Introduction

Lymphatic filariasis (LF) is a neglected tropical disease (NTD) caused by the nematode parasites *Wuchereria bancrofti, Brugia malayi* and *Brugia timori*. LF is transmitted by a variety of mosquito species.^1^ Infection can lead to chronic disabling, disfiguring and painful clinical symptoms, including lymphoedema (swelling of the limb) and hydrocoele (scrotal swelling).^2^ In addition, people affected by LF can experience stigma, social exclusion and discrimination that can have significant psychosocial consequences.^3^ An estimated 15 million people are living with filarial lymphoedema in marginalized communities in low- and middle-income countries.^1^ People with lymphoedema are prone to experiencing acute dermatolymphangioadenitis, also known as acute filarial attacks, which are secondary bacterial or fungal infections entering skin lesions, resulting in fever, chills, headache and localized inflammation in the involved region.^4,5^ Lymphoedema is a chronic condition that can dramatically impact an affected person and his/her family's capacity to work, reduce marital prospects and exacerbate poverty.^6–9^

Lymphoedema can predispose individuals to suffer from poor mental well-being, with the physical symptoms (pain, discomfort and reduced functioning) and social stigma and exclusion that an individual experiences contributing to mental distress.^10–13^ Individual experiences of stigma vary greatly depending on the cultural beliefs in affected communities and the severity of LF.^14^ Specific sociodemographic groups affected by LF have been found to have higher levels of depressive symptoms, including women, those suffering a longer duration of illness and those with more severe stages of lymphoedema or hydrocele,^15,16^ and a lower quality of life (QOL); disability and frequency and history of acute attacks has been found to be associated in other skin NTDs.^16^

Morbidity management and disability prevention (MMDP) is a crucial objective of the Global Programme to Eliminate Lymphatic Filariasis (GPELF) in order to alleviate the suffering of affected individuals.^2^ Malawi has successfully achieved elimination of LF as a public health problem by interrupting transmission with high coverage of mass drug administration (MDA) in all endemic regions as verified by the World Health Organization (WHO) in 2020. As many countries, including Malawi, complete MDA, their focus has shifted to the remaining individuals living with the debilitating symptoms of LF by providing the minimum package of care through MMDP.^17^ Implementation of MMDP is also an important facet of the WHO 2030 roadmap target of achieving a 75% reduction in global morbidity attributable to NTDs. For chronic limb lymphoedema in particular, the provision of MMDP care involves sustained, lifelong self-care treatment to delay progression or reverse lymphoedema symptoms and reduce acute filarial attacks through hygiene and skin exercises.^1^ Consequently, sustained lifelong care for lymphoedema management brings about challenges of adherence and sustainability, such as the allocation of health system resources and staff training for continued care.^18,19^ Short-term studies investigating self-care interventions, including augmented ‘enhanced’ self-care treatment for lymphoedema, which additionally includes deep-breathing exercises, lymphatic massage and dietary changes, have shown improved lymphoedema status and a significant reduction in the frequency and duration of acute filarial attacks.^20,21^ However, the indirect impact on the mental well-being of filarial lymphoedema sufferers following implementation of an enhanced self-care package is yet to be explored.

Mental well-being has been highlighted as a priority area within the new agenda for the WHO NTD roadmap 2021–2030, stating that mental health, psychosocial support and stigma reduction should be prioritized within NTD programs.^22^ There remains a lack of prioritization at the policy level regarding mental well-being support for people affected by NTDs.^3^ Therefore, an increased evidence base is required to determine the burden of mental health, identify risk factors and understand how available MMDP treatment impacts individuals’ well-being within endemic countries. In particular, as programs prioritize morbidity management activities, it is important to understand the effect on individuals’ mental well-being and if additional support is required for integration into programmatic activities.

There are several tools that can be used to assess this, including the 9-item Patient Health Questionnaire (PHQ-9) for measuring depressive symptoms and the LF-specific QOL Questionnaire (LFSQQ) for measuring QOL.^23,24^ The PHQ-9 tool is widely used as a depressive symptom screening tool in primary healthcare settings in high- and low-income countries and has been previously used in Malawi to assess diabetes patients.^25,26^ The LFSQQ has been used to measure the impact of self-care intervention compared against other QOL assessment tools and tested in a larger patient population.^24,27,28^

Therefore, this study aimed to determine the prevalence of key mental health indicators and depressive symptoms, poor QOL, the associated sociodemographic and clinical risk factors and the impact of an enhanced self-care intervention for lymphoedema management on depressive symptoms and QOL among affected individuals in Malawi.

## Methods

### Study site and design

**Figure 1.**
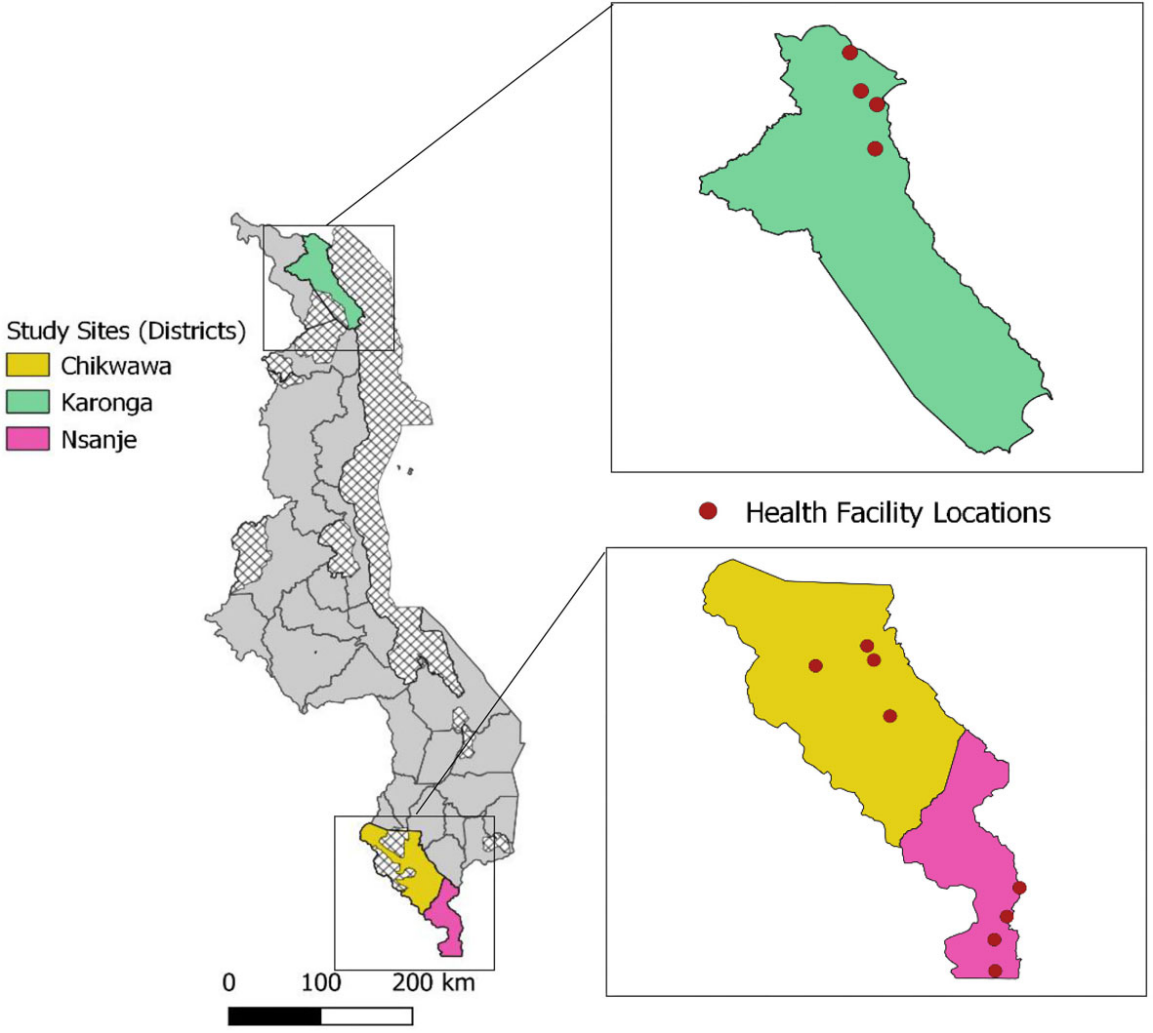
Study sites in Karonga (Northern), Chikwawa and Nsanje (Southern) districts and locations of the health facilities where the study surveys were conducted.

A prospective cohort study was conducted in three sites in Malawi between January and August 2021. The study was conducted in the Karonga (northern), Chikwawa and Nsanje (southern) districts in Malawi (Figure 1), historically known for their high endemicity of LF infection and high number of reported cases.^29,30^

This study was part of a larger study investigating the physical impact of the enhanced self-care intervention (paper in preparation). Adults (>18 y of age) affected by lower limb lymphoedema were identified from national programmatic records (based on house-to-house case mapping conducted in 2014, 2015 and 2017) and invited to participate in the study via the health centres. Baseline, 3- and 6-month follow-up survey's were conducted that included a range of questions on the sociodemographics, clinical characteristics and quality of life of people affected by lymphoedema.

The sample size was derived to observe changes in lymphoedema based on the results of a clinical trial.^21^ Within each study location, mild cases and moderate–severe lymphoedema cases were recruited, with staging defined according to the WHO lymphoedema staging criteria.^31^ In Malawi, we anticipated cases would be 75–79% female and 21–25% male based on the programmatic mapping [unpublished data].^32^ The sample size power was set at 85% and a significance level of 5% with Bonferroni correction for multiple testing, which was estimated to be 58 participants per group. The sample size was increased by 25% to allow adjustment for cofounders and loss to follow-up, yielding a final sample size of 73 participants per group (mild and moderate–severe), for a total of 146.

This study was conducted during intervals of the coronavirus disease 2019 (COVID-19) pandemic, with risk assessments conducted before any field work was implemented. All national guidelines and protocols for COVID-19, in accordance with the Ministry of Health, Malawi, were followed.

### Survey information and risk factors

Surveys included questions on the participant's sociodemographics (age, gender, geographic region, days taken off work), lymphoedema care history (requiring a caregiver for lymphoedema care, no caregiver), lymphoedema clinical characteristics (staging, number of affected legs, self-reported number of acute filarial attacks in the last 30 d) and mental health status (depressive symptoms, QOL). Acute filarial attacks were described to participants as an episode of acute inflammation, pain, redness and swelling in a limb with associated lymph nodes (kernels) and ague or fever.

Lymphoedema staging was assessed by an experienced field team member using the WHO lymphoedema staging criteria.^31^ Participants were categorized into two groups: mild staging, where both legs were categorized as WHO lymphoedema stage ≤1, or moderate–severe staging, where one or more legs were categorized as WHO lymphoedema stage ≥2.

### Enhanced self-care intervention

The surveys were conducted by experienced national health field teams, who were responsible for the training of participants in the enhanced self-care intervention to manage lymphoedema symptoms. This training included a combination of the WHO minimum care package^17^ and the enhanced self-care intervention.^20^ The standard WHO intervention included hygiene and skin care practices and daily and overnight elevation of the affected limb(s) and the enhanced self-care included deep-breathing techniques, lymphatic massage, skin mobilization, exercises (seated, standing and 45 min of walking per day) and eating fresh fruit and vegetables.

Of note, previous MMDP training in the WHO recommended package of care had been carried out by the community health workers when lymphoedema cases were first identified in 2014, 2015 (Chikwawa, Nsanje) and 2017 (Karonga), however, no specific follow-up had been conducted to determine adherence and impact.

### Tools to assess mental health

The PHQ-9 and the LFSQQ were used to assess mental health indicators: depressive symptoms and QOL, respectively.^23,24^ A Likert scoring system was used to collect questionnaire answers, with PHQ-9 scores ranging from 0 to 27 and LFSQQ scores ranging from 0 to 100.^33^ The PHQ-9 scores were defined according to widely used categories, including once previously in Malawi,^25^ as follows: no depressive symptoms (0–4), mild depressive symptoms (5–9), moderate depressive symptoms (10–14), moderately severe depressive symptoms (15–19) and severe depressive symptoms (20–27), and similarly for the LFSQQ: high QOL (0–29), moderately low QOL (30–49) and low QOL (50–100).^34,35^

### Data management

All survey data were collected and monitored in real time using the Open Data Kit Collect (ODK) software and uploaded via electronic tablets (Samsung Galaxy 10). Data were downloaded as a CSV file and imported into R programming for descriptive and statistical analysis (R Foundation for Statistical Computing, Vienna, Austria).^36^ Missing data were excluded from the final analysis.

#### Overview of statistical analysis

We conducted three separate analyses: descriptive, risk factor and longitudinal. Descriptive analysis was performed for sociodemographic factors, lymphoedema clinical characteristics, lymphoedema care and outcome variables, depressive symptoms and low QOL. Risk factors for depressive symptoms and low QOL were identified through a model selection process using univariable beta regression models. Longitudinal analysis investigated changes in depressive symptoms and QOL scores over the study time period to assess the sustained effect of the enhanced self-care intervention.

#### Risk factor analysis

Depressive symptoms and QOL were transformed to be bound between 0 and 1 following $Y^{\prime} = Y/N$, where $Y^{\prime}$ is the transformed score, $Y$ is the untransformed observed score and $N$ is the maximum possible score for each metric: 27 for the PHQ-9 (depressive symptoms) and 100 for the LFSQQ (QOL).

Candidate sociodemographic risk factors were age, gender, geographic region, requiring caregiver support and number of days off work in the past 30 d. Candidate clinical risk factors were lymphoedema staging, number of affected legs and the number of acute attacks in the past 30 d. Previous work on LF clinical conditions and QOL helped to guide the selection of candidate variables.^7,8,20^

Univariable logistic beta regression was performed on all candidate risk factors and then a backwards stepwise selection process was conducted to generate a multivariable model for each outcome variable. Candidate risk factor odds ratios (ORs), 95% confidence intervals (CIs) and p-values were calculated.

An additional post hoc model was fitted to data from participants with moderate–severe lymphoedema to explore the relationship between QOL and the number of affected legs. This model also included gender, requiring a caregiver and the number of days off work as additional variables.

#### Longitudinal analysis

As the study has no control group, we used a longitudinal model to assess whether a change in depressive symptoms or QOL score was associated with a secular time trend (seasonality) and time since the start of intervention using mixed effect beta regression models. To assess the temporal variation of observed follow-ups, the number of participants surveyed across secular time was plotted by the date of follow-up.

Beta regression models were fitted for depressive symptoms and QOL scores. The difference in score between the baseline and subsequent follow-up for each participant was transformed as follows:


\begin{eqnarray*}
{Y}_i = \left( {{S}_i - {S}_0} \right)/2N + 0.5,
\end{eqnarray*}

where *S*_0_ is the baseline score, *S_i_* is the score at follow-up *i* and *N* is the maximum score for each metric: 27 for the PHQ-9 (depressive symptoms) and 100 for the LFSQQ (QOL). The transformed score difference, *Y_i_*, rendered values between 0 and 1, where no change in score between baseline and follow-up would give *Y_i_* = 0.5.

Explanatory variables in the models included secular time (number of days since the start date of the study), time since baseline (number of days since the participant completed the baseline survey) and participant score at baseline. A random intercept was included for each individual. In the absence of a control group, including secular time and time since baseline allowed us to assess if any change in score could be attributed to the intervention rather than a general trend in the population. Baseline participant scores were included on the assumption that each participant’s follow-up scores were more likely to show a trend with their baseline score independent of other participants.

### Ethical approval

Ethical approval was obtained from the Liverpool School of Tropical Medicine, Research Ethics Committee, UK (research protocol 20-008) and National Health Sciences Research Committee, Malawi (2615). Participants received information sheets describing the study aims and data collection before they provided written consent.

## Results

The results from this study are organized into three sections. First, we report on the sociodemographic, lymphoedema care and clinical characteristics of the study participants. Second, we report the prevalence of depressive symptoms and QOL and identify risk factors associated with each of these outcomes. Finally, we determine changes in depressive symptoms and QOL following the intervention with a longitudinal analysis.

### Sociodemographic and clinical characteristics

A total 311 study participants completed baseline surveys across two regions in Malawi (Table 1). Participants were from Karonga (n=142 [45.8%]), Chikwawa (n=76 [24.5%]) or Nsanje (n=92 [29.7%]). As a result of challenges in participant recruitment during the response to COVID-19, adjacent study areas Chikwawa and Nsanje were combined to reach a sample size suitable for analysis. More than two-thirds of the study participants were female (68.1%) and the mean age of study participants was 56 y (range 18–90, standard deviation 14.3). Participants were from the Northern (144 [45.8%]) or Southern (168 [54.2%]) regions of Malawi, shown in Figure 1. Of the study participants, 68 (21.9%) reported requiring a caregiver. Most participants described their main source of financial support (employment) to be from farming (242 [77%]) or family support (42 [13.5%]). The majority of participants (194 [62.4%]) reported no days off work in the last 30 d due to illness. The remaining participants required 1–7 d (76 [24.4%]), 8–14 d (29 [9.3%]) and ≥15 d (12 [3.9%]) off work.

**Table 1.** Sociodemographic, clinical symptoms and well-being assessment of the study participants (N=311)

Socio-demographic Factors	n (%)
Gender	
Female	212 (68.2)
Male	99 (31.8)
Age (years)	
18–29	10 (3.2)
30–39	24 (7.7)
40–49	73 (23.5)
50–59	82 (26.4)
60–69	64 (20.6)
≥70	54 (17.4)
Geographic region	
Northern	144 (46.0)
Southern	168 (54.0)
District	
Karonga	144 (46.0)
Chikwawa	73 (24.4)
Nsanje	92 (29.6)
Requires caregiver	
Yes	243 (78.1)
No	68 (21.9)
Employment (job)	
Farmer	242 (77.8)
Relies on family support	42 (13.5)
Other	27 (8.7)
Days off work (in the last month)	
None	194 (62.4)
1–7	76 (24.4)
8–14	29 (9.3)
≥15	12 (3.9)
Clinical symptoms	
Lymphoedema staging	
Mild	104 (33.4)
Moderate–severe	207 (66.6)
Staging ≥2 present	
Both legs	276 (88.7)
One or no legs	35 (11.3)
Acute attacks in the last month	
0	139 (44.7)
1	146 (46.9)
2	19 (6.1)
≥3	7 (2.3)
Well-being assessment	
Depression (PHQ-9 score)	
No depression (<5)	238 (76.5)
Mild (≥5–<10)	63 (20.3)
Moderate (≥10–<15)	10 (3.2)
Moderately severe (≥15)	0 (0.0)
QOL (LFSQQ score)	
Not low (<30)	213 (68.5)
Moderately low (≥30–<50)	89 (28.6)
Low (≥50)	9 (2.9)

The clinical characteristics assessed included lymphoedema staging: 104 (33.4%) were mild and 207 (66.6%) were moderate–severe. The number of individuals who had a WHO lymphoedema staging of ≥2 on both legs was 35 (11.3%). The number of participants who had experienced no acute attacks in the last month was 139 (44.7%), a single acute attack 146 (46.9%), two attacks 19 (6.1%) and three or more attacks was 7 (2.3%).

**Figure 2.**
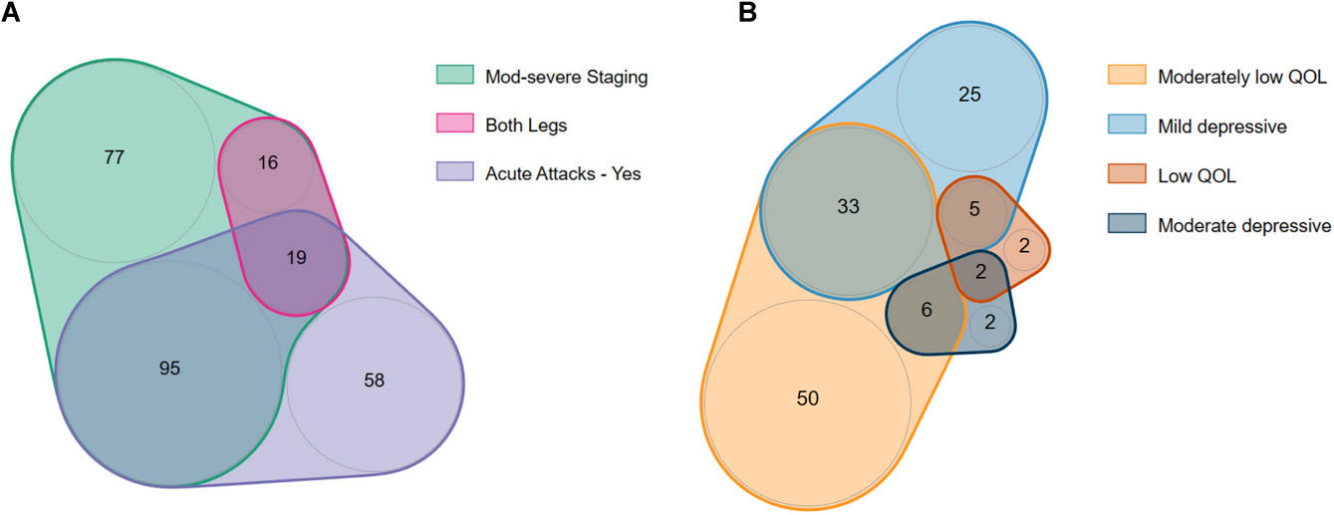
Venn diagrams presenting **(A)** clinical symptoms and **(B)** depressive symptoms and low QOL of participants. Note. Moderate-severe lymphoedema staging (displayed on one or both legs), both legs displaying moderate-severe staging and having experienced an acute attack in the last month, to show the number of participants exhibiting described symptoms. Mild depressive symptoms, moderate depressive symptoms, moderately low QOL and low QOL to show the number of participants exhibiting mental health indicators.

Overlapping proportions of study participants with most severe clinical symptoms—moderate–severe lymphoedema, both legs displaying moderate–severe lymphoedema and experience of an acute attack in the last month—are represented as a Venn diagram in Figure 2a. Overlapping proportions of study participants with depressive symptoms and low QOL—mild depressive symptoms, moderate depressive symptoms, moderately low QOL and low QOL—are represented as a Venn diagram in Figure 2b.

### Mental health baseline analysis

At baseline, of 311 patients, 20.3% (95% CI 15.9 to 25.2) reported mild depressive symptoms and 3.2% (95% CI 1.5 to 5.8) reported moderate depressive symptoms. For QOL, 28.6% (95% CI 23.7 to 34.0) reported moderately low QOL and 2.9% (95% CI 23.7 to 34.0) reported severely low QOL.

#### Depressive symptoms risk factor analysis

**Figure 3.**
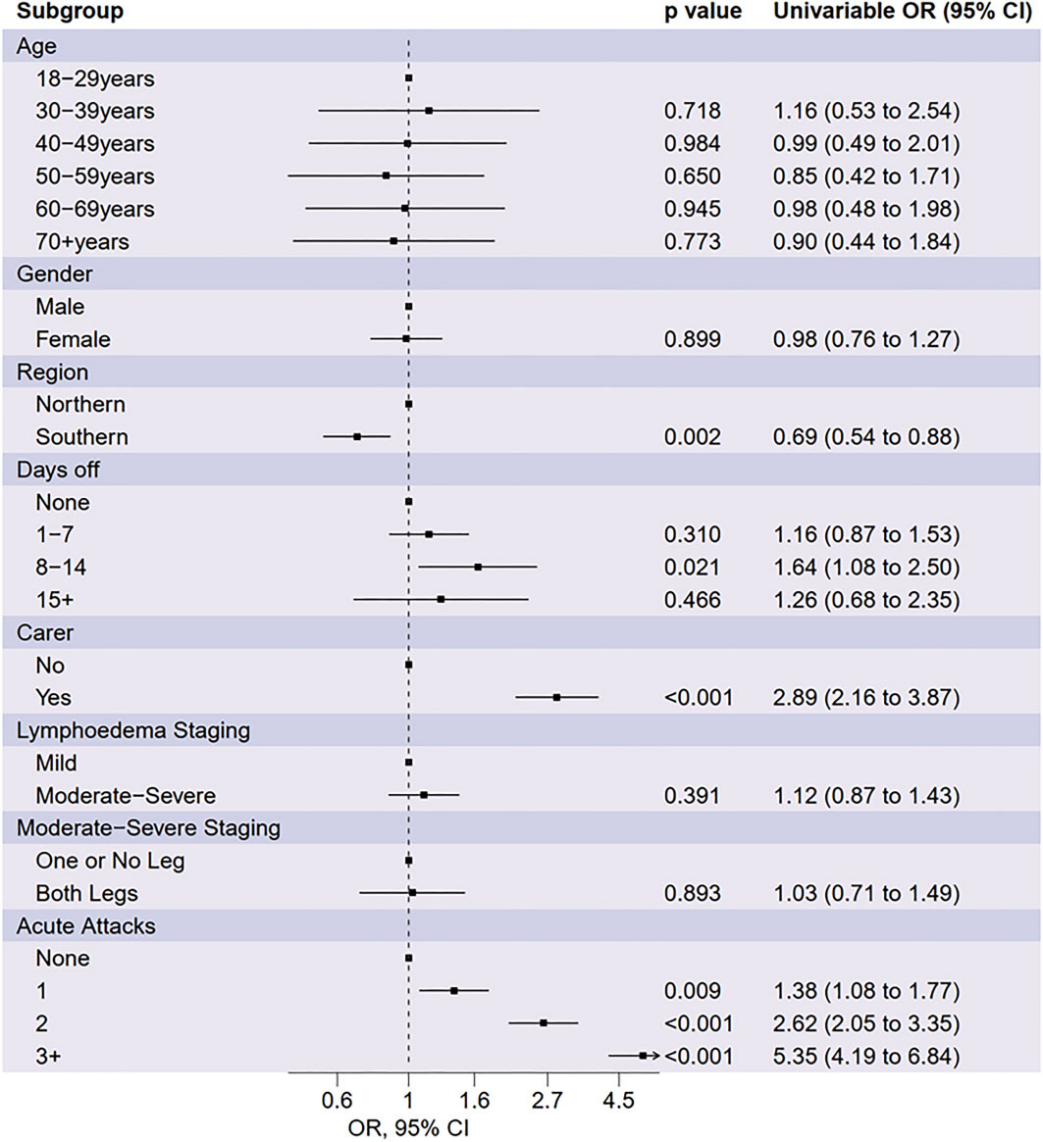
ORs and 95% CIs for depressive symptoms derived from univariable beta regression models.

Figure 3 shows estimated ORs, 95% CIs and p-values for univariable analysis of all candidate risk factors. Participants located in the Southern region of Malawi (OR 0.69 [95% CI 0.54 to 0.88]) were associated with lower depressive symptoms scores. Participants who required a caregiver (OR 2.89 [95% CI 2.16 to 3.87]) and had taken 8–14 d off work due to LF in the last 30 days (OR 1.64 [95% CI 1.08 to 2.50]) were associated with higher levels of depressive symptoms. Of the candidate clinical risk factors, the frequency of acute attacks in the last month (one acute attack, OR 1.38 [95% CI 1.08 to 1.77]; two acute attacks, OR 2.62 [95% CI 2.05 to –3.35]; three or more acute attacks, OR 5.32 [95% CI 4.19 to 6.84]) was associated with higher depressive symptom scores. Moderate–severe staging was not found to be associated with depressive symptoms.

#### Depressive symptoms multivariable model

**Figure 4.**
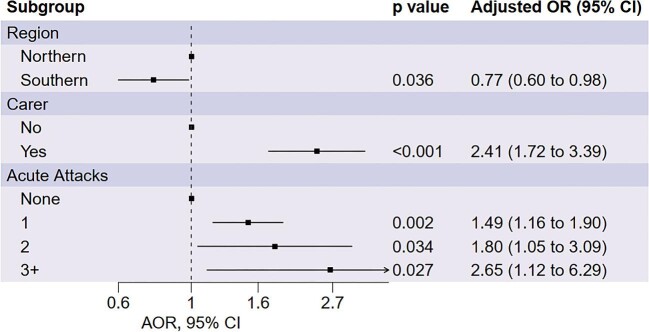
AORs and 95% CIs for depressive symptoms derived from a multivariate model selected through backwards stepwise selection.

Following a backward stepwise selection process, we found that the frequency of acute attacks, requiring a caregiver and geographic region were association with depressive symptoms scores, shown in Figure 4. We found that Southern participants were associated with lower depressive symptoms scores (adjusted OR [AOR] 0.77 [95% CI 0.60 to 0.98]). A higher frequency of acute attacks in the last month (one acute attack, aOR 1.49 [95% CI 1.16 to 1.90]; two acute attacks, aOR 1.80 [95% CI 1.05 to 3.09]; three of more acute attacks, aOR 2.65 [95% CI 1.12 to 6.29]) and requiring a caregiver (aOR 2.41 [95% CI 1.72 to 3.39) were associated with higher levels of depressive symptoms.

#### QOL risk factor analysis

**Figure 5.**
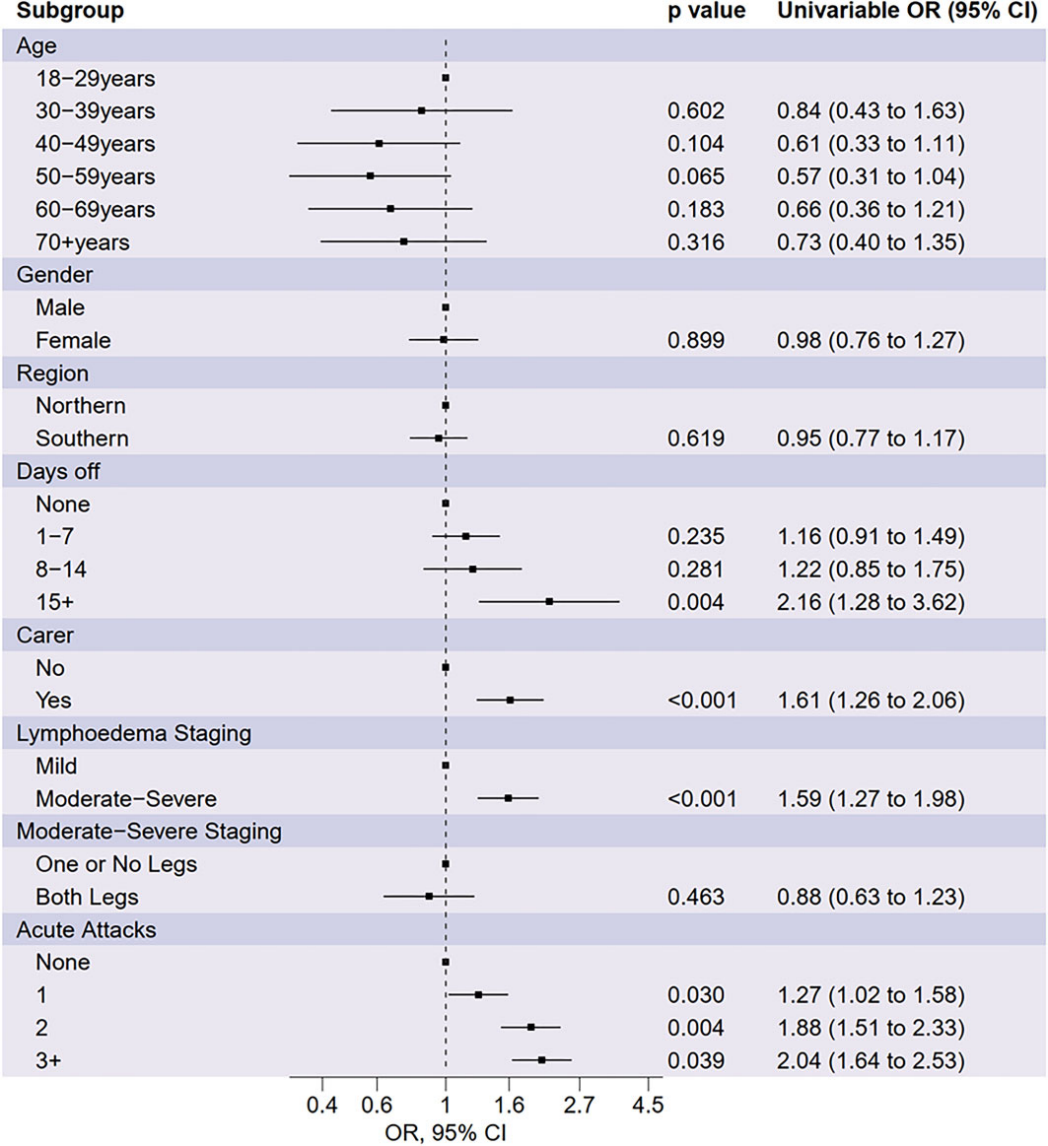
ORs and 95% CIs for QOL derived from univariable beta regression models.

Figure 5 shows the estimated ORs, 95% CIs and p-values for univariable analysis of all candidate risk factors. Participants who required a caregiver (OR 1.16 [95% CI 1.26 to 2.06]) and had ≥15 d off work in the last 30 d (OR 2.16 [95% CI 1.28 to 3.62]) were associated with lower QOL. Of the candidate clinical risk factors, moderate–severe lymphoedema staging (OR 1.59 [95% CI 1.27 to 1.98]) and frequency of acute attacks in the last month (one acute attack, OR 1.27 [95% CI 1.02 to 1.58]; two acute attacks, OR 1.88 [95% CI 1.51 to 2.33]; three or more acute attacks, OR 2.04 [95% CI 1.68 to 2.54]) was associated with lower QOL.

#### QOL multivariable model

**Figure 6.**
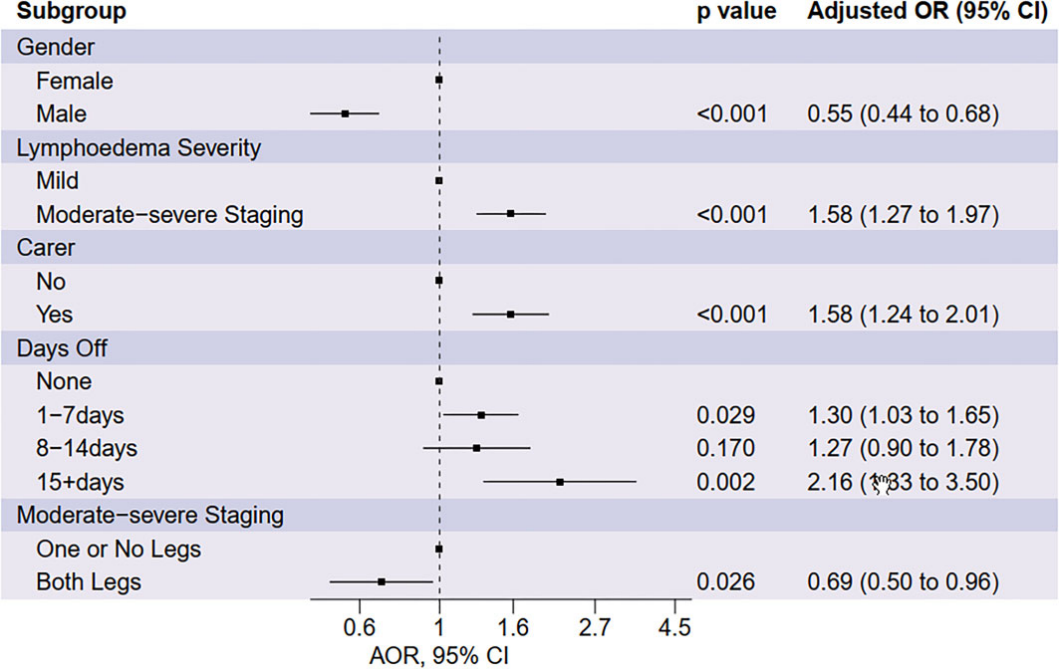
AORs for QOL derived from a multivariate model selected through backwards stepwise selection.

Following a backward stepwise selection process, we found that gender, lymphoedema staging, requiring a caregiver, number of days off work and moderate–severe staging on both legs were associated with QOL scores, shown in Figure 6. We found that moderate–severe staging of lymphoedema (AOR 1.58 [95% CI 1.27 to 1.97]) associated with lower QOL. From candidate sociodemographic risk factors, requiring a caregiver (OR 1.58 [95% CI 1.24 to 2.01]) and taking days off work (1–7 d, OR 1.30 [95% CI 1.03 to 1.65]; ≥15 d, OR 2.16 [95% CI 1.33 to 3.50]) were associated with lower QOL. Being male (OR 0.55 [95% CI 0.44 to 0.68]) and having moderate–severe lymphoedema on both legs (OR 0.69 [95% CI 0.50 to 0.96]) was associated with higher QOL. To further explore the results from this model that participants with moderate–severe lymphoedema staging on both legs had improved QOL, we fitted an additional model to the subset of participants with moderate–severe lymphoedema staging and found a significant association with both legs being affected.

### Longitudinal analysis

Longitudinal survey data were collected across the three districts over a period of 9 months (see Supplementary Figure 1). Baseline surveys and 6-month follow-ups were collected in the Northern region in January and July and in the Southern region in March and August 2021. Southern districts were delayed due to challenges with COVID-19 and the Ministry of Health response. A total of 311 participants completed the baseline survey, 284 completed the 3-month follow-up survey and 276 completed the 6-month follow-up survey.

In the absence of a control group, we fitted beta regression models to identify the association of secular time, time since the start of intervention and baseline score with changes in depressive symptoms and QOL. We found a significant intercept term and a significant association between baseline score and change in depressive symptoms score, however, we did not find any association between secular time or time since the start of intervention and the change in depressive symptoms score (Table 2). The interpretation of these findings is that individuals with a baseline depressive symptoms score of 0 tended to have a higher score at the first follow-up and individuals with a baseline depressive symptoms score >0 tended to have lower depressive symptoms scores at the first follow-up (Table 3). Scores remained unchanged at the 6-month follow-up. As there was no significant association with time since intervention and change in depressive symptoms score, there was no direct evidence to suggest that the intervention was associated with a reduction in depressive symptoms.

**Table 2.** Odds ratio (OR) of change in depressive symptom (PHQ-9) and quality of life (LFSQQ) scores

	Depression (PHQ-9)		Quality of life (LSFQQ)	
Fixed effects variables	OR (95% CI)	p-Value	OR (95% CI)	p-Value
Intercept	**1.06 (1.02 to 1.10)**	<0.001	**1.22 (1.17 to 1.28)**	<0.001
Score at baseline	**0.93 (0.93 to 0.93)**	<0.001	**0.98 (0.98 to 0.98)**	<0.001
Secular time	1.00 (1.00 to 1.00)	0.264	**0.99 (0.99 to 0.99)**	0.0196
Time since baseline	1.00 (1.00 to 1.00)	0.853	0.99 (0.99 to 1.00)	0.3155

Variables significant at the 95% CI level are shown in bold, with 23.2% and 23.1% of the variance in outcome variables being explained by the random effect in the depressive symptoms and QOL models, respectively.

**Table 3.** Predicted scores using beta regression models with average time since intervention at the 3- and 6-month follow-ups and the average secular time at the 3- and 6-month follow-ups

Depression (PHQ-9)^a^			Low QOL (LFSQQ)^b^		
Baseline	Predicted score at 3	Predicted score at 6	Baseline	Predicted score at 3	Predicted score at 6
score	months (95% CI)	months (95% CI)	score	months (95% CI)	months (95% CI)
0	0.2 (0.1 to 0.3)	0.2 (0.0^c^ to 0.5)	0	2.7 (2.0 to 3.5)	1.0 (0.0^c^ to 2.4)
1	0.7 (0.6 to 0.9)	0.7 (0.4 to 1.0)	2	3.8 (3.2 to 4.5)	2.1 (0.7 to 3.4)
2	1.3 (1.2 to 1.4)	1.2 (0.9 to 1.6)	3	4.4 (3.7 to 5.1)	2.6 (1.2 to 4.0)
3	1.8 (1.7 to1.9)	1.8 (1.4 to 2.1)	4	4.9 (4.3 to 5.6)	3.2 (1.8 to 4.5)
4	2.3 (2.2 to 2.4)	2.3 (2.0 to 2.6)	5	5.5 (4.9 to 6.1)	3.7 (2.3 to 5.1)
5	2.9 (2.7 to 3.0)	2.8 (2.5 to 3.1)	6	6.0 (5.4 to 6.7)	4.3 (2.9 to 5.6)
6	3.4 (3.3 to 3.5)	3.3 (3.0 to 3.7)	8	7.1 (6.6 to 7.7)	5.4 (4.0 to 6.7)
7	3.9 (3.8 to 4.1)	3.9 (3.6 to 4.2)	10	8.2 (7.7 to 8.8)	6.5 (5.1 to 7.8)
8	4.5 (4.3 to 4.7)	4.4 (4.1 to 4.8)	12	9.3 (8.8 to 9.9)	7.6 (6.2 to 8.9)
9	5.0 (4.9 to 5.2)	5.0 (4.7 to 5.3)	14	10.5 (10.0 to 10.9)	8.7 (7.4 to 10.0)
10	5.6 (5.4 to 5.8)	5.6 (5.2 to 5.9)	16	11.6 (11.1 to 12.0)	9.8 (8.5 to 11.1)
11	6.2 (6.0 to 6.4)	6.2 (5.8 to 6.5)	18	12.7 (12.2 to 13.1)	10.9 (9.6 to 12.2)
12	6.8 (6.6 to 7.0)	6.7 (6.4 to 7.1)	20	13.8 (13.3 to 14.2)	12.0 (10.7 to13.3)
13	7.4 (7.1 to 7.6)	7.3 (7.0 to 7.7)	22	14.9 (14.5 to 15.3)	13.2 (11.9 to 14.5)
14	8.0 (7.7 to 8.2)	8.0 (7.6 to 8.3)	24	16.0 (15.6 to 16.4)	14.3 (13.0 to 15.6)
15	8.6 (8.4 to 8.9)	8.6 (8.2 to 8.9)	26	17.1 (16.7 to 17.6)	15.4 (14.1 to 16.7)

^a^0: not depressed, 27: highest depression.

^b^0: best QOL, 100: lowest QOL.

c CIs were truncated at 0.

We found a significant intercept term and a significant association between baseline score and secular time and change in QOL score and no association between time since intervention and change in QOL score (Table 3). Individuals with a baseline QOL score <5 tended to have a higher score at the first follow-up (Table 3). The interpretation of these findings is that individuals with a baseline QOL score <5 tended to have a higher score at the 3-month follow-up, individuals with baseline QOL scores >5 tended to have improved QOL scores at the first follow-up and generally all individual scores decreased from the 3-month to 6-month follow-up (Table 3). We found that secular time was associated with a change in QOL, therefore, as time continued throughout the study, the QOL generally improved. However, there was no direct evidence that the intervention was associated with an improvement of QOL, as there was no significant association with time since intervention and change in QOL.

## Discussion

This is the first study to look at the prevalence of mental health indicators in people affected by lymphoedema in Malawi and associated risk factors. Previous literature exploring the mental health of people affected by filariasis found the prevalence of depressive symptoms ranged dramatically depending on the region of the world, e.g. in Africa prevalence ranged from 20 to 70% and in Asia it ranged from 8.5 to 37%.^13,16,37–41^ Studies measuring QOL among people affected by filarial lymphoedema found significantly lower QOL scores using different assessment tools.^42–46^

In high-income countries, there is substantial evidence to show that people living with a physical disability have at least a three times greater chance of experiencing depression compared with the general population.^47–49^ Disability has been found to be associated with higher levels of depression in LF and other skin NTDs.^16,50^ This study found that higher levels of depressive symptoms in lymphoedema sufferers were associated with a higher number of acute attack episodes in the last month, similar to findings in Bartlett et al. investigating podoconiosis.^50^ Acute attack episodes have been described as a contributor to the severity of disability of LF at a specific time point, as opposed to ongoing permanent disability, particularly as acute attacks hinder mobility and the ability to practice self-care and work, which results in lost earnings.^44^ In addition, the frequency, intensity and duration of acute attacks result in much of the LF burden for affected persons and their communities.^51,52^; e.g. caregivers were found to have significantly lower QOL while providing care during an acute attack episode.^7,53,54^ Consequently, individuals experiencing acute attacks require more care and treatment to stop the progression of disease and damage to lymphatics and alleviate challenges to mental well-being and incomes in affected communities.^5^

In this study, two-thirds of the participants with lymphoedema were women. In Malawi, LF-related lymphoedema disproportionately effects women, and this has been shown in multiple other countries,^32,55,56^ including in Bangladesh, where 3.7 times more women were found to have lymphoedema than men.^57^ In addition, the caregivers for people with lymphoedema are more often women,^54^ showing that the burden of lymphoedema falls disproportionately on women. Having a greater proportion of women may have impacted the results of this study, particularly as women have been found to be associated with a higher prevalence of depression in low- and middle-income countries.^58^ We found that participants with moderate–severe lymphoedema on both legs were associated with having a higher QOL; however, this may be due to an unbalanced study design, because all participants with both legs affected had moderate–severe lymphoedema. To explore this further we refitted the model with only participants displaying moderate–severe lymphoedema and found a significant association between higher QOL and having both legs affected with moderate–severe lymphoedema. This was an unexpected result for which we have no explanation; it may be an apparent association generated by an unmeasured confounding variable.

This longitudinal study demonstrates that lymphoedema self-care may play a role in improving depression and QOL within the study cohort. When changes in mental health indicators were modelled, we found that individuals with higher depressive symptoms and lower QOL scores at baseline were associated with greater improvements in scores. Generally, all individuals experienced an improvement in QOL over the study time period. However, there was no significant improvement in QOL related to time since the start of the enhanced self-care intervention, so we would conclude this change is most likely not directly related to the intervention and rather a population-scale phenomenon associated with a factor we did not measure. We found there was no direct evidence to suggest that the lymphoedema enhanced self-care intervention was associated with improved mental health indicators, despite significant and clinically relevant improvements in lymphoedema being observed.^21^ To assess if an improvement in depressive symptoms and QOL scores was maintained after the study will require additional follow-ups.

One reason why this study found no direct evidence to suggest that the lymphoedema enhanced self-care intervention improved mental health indicators could be related to the lack of mental health interventions. Other NTD research has highlighted that an integrated self-care and mental health intervention can improve physical symptoms, QOL and depression.^27,59^ Depression and NTDs alone can cause drastic health consequences^60,61^ and their comorbidity is likely to further exacerbate such consequences. There is growing evidence that improved mental health support and NTD services reduce the risk of both morbidities.^50^ Therefore, the addition of mental health support within the minimum care package for people affected by LF would further alleviate suffering.

It is important to note that prior to this study, treatment was not available in health services in Malawi for people affected by filarial lymphoedema, other than the prescription of painkillers during acute attack episodes and one-off WHO minimum package of care training for lymphoedema management.^17^ Therefore, participants receiving a more in-depth enhanced self-care training, as well as enrolment into this study, which included the provision of medical supplies to practice self-care, may have played a role in contributing to their improved mental well-being.^62^

The findings from this study highlight the importance of widening the scope of LF morbidity management to provide psychosocial support for affected persons as well as physical morbidity management.^44^ The long-lasting psychosocial problems associated with lymphoedema could mean that lymphoedema management alone may be insufficient in addressing the psychosocial implications for affected individuals, their families and caregivers.^3^ Currently, support and management for mental well-being among persons affected by LF is limited except for specific non-governmental organizations or research work.^63–65^

Ideally, adopting a holistic approach that includes psychological support such as counselling (e.g. by Mental Health Gap Action Programme–trained clinicians), peer support groups and referral to tertiary health systems; education of LF stigma reduction interventions in affected communities and implementation of morbidity management for physical symptoms, such as self-care for lymphoedema and hydrocele surgeries; and rehabilitation and economic support. Integration of these activities within health systems or available services will play an important role in improving their sustainability.^66^ Providing affected persons with a sustained person-centred care program will not only have psychological and behavioural benefits, but will also help to optimize skin care self-management observed in other lymphoedema research.^67^

### Limitations

One limitation of this study was the absence of a control group, meaning firm conclusions about the effectiveness of the intervention could not be made, although we did look at secular time as a confounder for the effects we do see and did not find any effect for depressive symptoms. Longitudinal analysis is limited by the clustering of observations in time, making it difficult to distinguish between any effect of secular time and time since intervention on outcome variables. Some sampling bias in collecting of mild and moderate–severe cases may limit the generalizability of risk factor analysis. There is limited literature on the validity, cultural interpretability and use of the PHQ-9 in Africa, particularly in Malawi. Specifically, cultural interpretability is important when using questionnaires developed for assessment within another country or context, potentially introducing measurement bias and underestimating the prevalence of mental health indicators within this study group. The lack of 2-week repeats of the PHQ-9 assessments is a limitation of the study, meaning depression could not be diagnosed as according to the Diagnostic and Statistical Manual of Mental Disorders, 4th edition criteria, therefore the authors referred to the PHQ-9 result as ‘depressive symptoms’. Some external factors, such as those with a history of family illness, wealth index or other comorbidities, that may impact on depressive symptoms and low QOL in participants were not measured. The tools used to assess depressive symptoms and QOL, thePHQ-9 and LFSQQ, were conducted as part of an extensive survey (107 questions) that could have introduced participant fatigue and may have impacted the reliability of scores. The longitudinal model is not intended as a clinical prediction tool, as we modelled the change in score rather than the absolute score itself.

## Conclusions

This study's findings indicate that mental health indicators, depressive symptoms and low QOL, are prevalent in people affected by lymphoedema within this Malawi population. The enhanced self-care intervention may have played a role in improving depressive symptoms and QOL among participants, with individuals with higher depressive symptoms and lower QOL showing the greatest improvements. However, there was no direct evidence to suggest that the lymphoedema enhanced self-care intervention was associated with improved mental health indicators. Therefore, as well as providing effective morbidity management to alleviate suffering from physical symptoms of lymphoedema, a more holistic approach including the provision of psychosocial support for affected persons is recommended.

## Supplementary Material

ihad064_Supplemental_Figure

## Data Availability

Regional and district data are summarised in paper. Individual level data can not be shared as it will compromise the privacy of individuals that participated in the study.

## References

[1] World Health Organization . Lymphatic filariasis: Key facts. Available from: https://www.who.int/news-room/fact-sheets/detail/lymphatic-filariasis [accessed 6 April 2023].

[2] World Health Organization . Global programme to eliminate lymphatic filariasis: progress report, 2021. Wkly Epidemiol Rec. 2022;97(41):513–24.

[3] Bailey F , Eaton J, Jidda M et al. Neglected tropical diseases and mental health: progress, partnerships, and integration. Trends Parasitol. 2019;35(1):23–31. 30578149 10.1016/j.pt.2018.11.001

[4] El-Nahas H , El-Shazly A, Abulhassan M et al. Impact of basic lymphedema management and antifilarial treatment on acute dermatolymphangioadenitis episodes and filarial antigenaemia. J Glob Infect Dis. 2011;3(3):227–32. 21887053 10.4103/0974-777X.83527 PMC3162808

[5] Shenoy RK . Clinical and pathological aspects of filarial lymphedema and its management. Korean J Parasitol 2008;46(3):119–25. 18830049 10.3347/kjp.2008.46.3.119 PMC2553332

[6] Mackenzie CD , Lazarus WM, Mwakitalu ME et al. Lymphatic filariasis: patients and the global elimination programme. Ann Trop Med Parasitol. 2009;103(Suppl 1):41–51. 10.1179/000349809X12502035776630 19843397

[7] Caprioli T , Martindale S, Mengiste A et al. Quantifying the socio-economic impact of leg lymphoedema on patient caregivers in a lymphatic filariasis and podoconiosis co-endemic district of Ethiopia. PLoS Negl Trop Dis. 2020;14(3):e0008058. 32126081 10.1371/journal.pntd.0008058 PMC7069637

[8] Martindale S , Chiphwanya J, Matipula DE et al. The wider societal benefits of surgical interventions for lymphatic filariasis morbidity management and disability prevention. PLoS Negl Trop Dis. 2021;15(9):e0009701. 34529661 10.1371/journal.pntd.0009701 PMC8445426

[9] Sawers L , Stillwaggon E, Chiphwanya J et al. Economic benefits and costs of surgery for filarial hydrocele in Malawi. PLoS Negl Trop Dis. 2020;14(3):e0008003. 32210436 10.1371/journal.pntd.0008003 PMC7094819

[10] Litt E , Baker MC, Molyneux D. Neglected tropical diseases and mental health: a perspective on comorbidity. Trends Parasitol. 2012;28(5):195–201. 22475459 10.1016/j.pt.2012.03.001

[11] Chapman DP , Perry GS, Strine TW. The vital link between chronic disease and depressive disorders. Prev Chronic Dis. 2005;2(1):A14. PMC1323317 15670467

[12] Turvey CL , Schultz SK, Beglinger L et al. A longitudinal community-based study of chronic illness, cognitive and physical function, and depression. Am J Geriatr Psychiatry. 2009;17(8):632–41. 19634203 10.1097/jgp.0b013e31819c498c PMC3690465

[13] Richard SA , Mathieu E, Addiss DG et al. A survey of treatment practices and burden of lymphoedema in Togo. Trans R Soc Trop Med Hyg. 2007;101(4):391–7. 17112555 10.1016/j.trstmh.2006.08.011

[14] Hofstraat K , van Brakel WH. Social stigma towards neglected tropical diseases: a systematic review. Int Health. 2016;8(Suppl 1):i53–70. 26940310 10.1093/inthealth/ihv071

[15] Abdulmalik J , Nwefoh E, Obindo J et al. Emotional difficulties and experiences of stigma among persons with lymphatic filariasis in Plateau State, Nigeria. Health Hum Rights. 2018;20(1):27–40. 30008550 PMC6039724

[16] Ali O , Deribe K, Semrau M et al. A cross-sectional study to evaluate depression and quality of life among patients with lymphoedema due to podoconiosis, lymphatic filariasis and leprosy. Trans R Soc Trop Med Hyg. 2020;114(12):983–94. 33190154 10.1093/trstmh/traa130 PMC7738660

[17] World Health Organization . Lymphatic filariasis: managing morbidity and preventing disability: an aide-mémoire for national programme managers. Geneva: World Health Organization; 2021.

[18] Suma TK , Shenoy RK, Kumaraswami V. Efficacy and sustainability of a footcare programme in preventing acute attacks of adenolymphangitis in Brugian filariasis. Trop Med Int Health. 2002;7(9):763–6. 12225507 10.1046/j.1365-3156.2002.00914.x

[19] Mackenzie CD , Mante S. Caring for patients in the global programme to eliminate lymphatic filariasis. Int Health. 2020;13(Suppl 1):S48–54. 33349884 10.1093/inthealth/ihaa080 PMC7753172

[20] Douglass J , Mableson HE, Martindale S et al. An enhanced self-care protocol for people affected by moderate to severe lymphedema. Methods Protoc. 2019;2(3):77. 31487887 10.3390/mps2030077 PMC6789820

[21] Douglass J , Mableson H, Martindale S et al. Effect of an enhanced self-care protocol on lymphedema status among people affected by moderate to severe lower-limb lymphedema in Bangladesh, a cluster randomized controlled trial. J Clin Med. 2020;9(8):2444. 32751676 10.3390/jcm9082444 PMC7464742

[22] World Health Organization . Ending the neglect to attain the sustainable development goals: a road map for neglected tropical diseases 2021–2030. Geneva: World Health Organization; 2020.

[23] Kroenke K , Spitzer RL, Williams JB. The PHQ-9: validity of a brief depression severity measure. J Gen Intern Med. 2001;16(9):606–13. 11556941 10.1046/j.1525-1497.2001.016009606.x PMC1495268

[24] Thomas C , Narahari SR, Bose KS et al. Comparison of three quality of life instruments in lymphatic filariasis: DLQI, WHODAS 2.0, and LFSQQ. PLoS Negl Trop Dis. 2014;8(2):e2716. 24587467 10.1371/journal.pntd.0002716 PMC3930502

[25] Udedi M , Muula AS, Stewart RC et al. The validity of the Patient Health Questionnaire-9 to screen for depression in patients with type-2 diabetes mellitus in non-communicable diseases clinics in Malawi. BMC Psychiatry. 2019;19(1):81. 30813922 10.1186/s12888-019-2062-2 PMC6391834

[26] Costantini L , Pasquarella C, Odone A et al. Screening for depression in primary care with Patient Health Questionnaire-9 (PHQ-9): a systematic review. J Affect Disord. 2021;279:473–83. 33126078 10.1016/j.jad.2020.09.131

[27] Aggithaya MG , Narahari SR, Vayalil S et al. Self care integrative treatment demonstrated in rural community setting improves health related quality of life of lymphatic filariasis patients in endemic villages. Acta Trop. 2013;126(3):198–204. 23499714 10.1016/j.actatropica.2013.02.022

[28] Budge PJ , Little KM, Mues KE et al. Impact of community-based lymphedema management on perceived disability among patients with lymphatic filariasis in Orissa State, India. PLoS Negl Trop Dis. 2013;7(3):e2100. 23516648 10.1371/journal.pntd.0002100 PMC3597476

[29] Nielsen NO , Makaula P, Nyakuipa D et al. Lymphatic filariasis in Lower Shire, southern Malawi. Trans R Soc Trop Med Hyg. 2002;96(2):133–8. 12055799 10.1016/s0035-9203(02)90279-8

[30] Ngwira BM , Jabu CH, Kanyongoloka H et al. Lymphatic filariasis in the Karonga district of northern Malawi: a prevalence survey. Ann Trop Med Parasitol. 2002;96(2):137–44. 12080974 10.1179/0003498302125000411

[31] World Health Organization . Lymphatic pathology and immunopathology in filariasis. Report of the Twelfth Meeting in Filariasis. TDR/FIL-SWG12, 85.3. Geneva: World Health Organization; 1985.

[32] Stanton MC , Mkwanda SZ, Debrah AY et al. Developing a community-led SMS reporting tool for the rapid assessment of lymphatic filariasis morbidity burden: case studies from Malawi and Ghana. BMC Infect Dis. 2015;15(1):214. 25981497 10.1186/s12879-015-0946-4 PMC4455607

[33] Likert R . A technique for the measurement of attitudes. Arch Psychol. 1932;22(140):55–155.

[34] Sebera F , Vissoci JRN, Umwiringirwa J et al. Validity, reliability and cut-offs of the Patient Health Questionnaire-9 as a screening tool for depression among patients living with epilepsy in Rwanda. PLoS One. 2020;15(6):e0234095. 32530968 10.1371/journal.pone.0234095 PMC7292570

[35] Asiedu SO , Kwarteng A, Amewu EKA et al. Financial burden impact quality of life among lymphatic filariasis patients. BMC Public Health. 2021;21(1):174. 33478462 10.1186/s12889-021-10170-8 PMC7818560

[36] R Development Core Team . A language and environment for statistical computing. Vienna: R Foundation for Statistical Computing; 2019.

[37] Obindo J , Abdulmalik J, Nwefoh E et al. Prevalence of depression and associated clinical and socio-demographic factors in people living with lymphatic filariasis in Plateau State, Nigeria. PLoS Negl Trop Dis. 2017;11(6):e0005567. 28570585 10.1371/journal.pntd.0005567 PMC5453421

[38] Dienye PO , Gbeneol PK, Akani AB. The association between giant hydrocele and depression in a rural clinic in Nigeria. Am J Mens Health. 2011;5(5):438–43. 21659355 10.1177/1557988311406981

[39] Mangeard-Lourme J , de Arguer GR, Parasa J et al. Depression and anxiety in people affected by leprosy and lymphatic filariasis: a cross-sectional study in four states in India. Lepr Rev. 2020;91(4):367–82.

[40] Wijesinghe RS , Wickremasinghe AR, Ekanayake S et al. Physical disability and psychosocial impact due to chronic filarial lymphoedema in Sri Lanka. Filaria J. 2007;6:4. 17391538 10.1186/1475-2883-6-4 PMC1851956

[41] Kanda K . The quality of life among lymphedema patients due to lymphatic filariasis in three rural towns in Haiti. Master's thesis, University of South Florida, 2004.

[42] Babu B , Nayak A, Rath K et al. Use of the Dermatology Life Quality Index in filarial lymphoedema patients. Trans R Soc Trop Med Hyg. 2006;100(3):258–63. 16289632 10.1016/j.trstmh.2005.05.022

[43] Chandrasena T , Premaratna R, Muthugala M et al. Modified Dermatology Life Quality Index as a measure of quality of life in patients with filarial lymphoedema. Trans R Soc Trop Med Hyg. 2007;101(3):245–9. 17098268 10.1016/j.trstmh.2006.08.012

[44] Kumari A , Harichandrakumar K, Das L et al. Physical and psychosocial burden due to lymphatic filariasis as perceived by patients and medical experts. Trop Med Int Health. 2005;10:567–73. 15941420 10.1111/j.1365-3156.2005.01426.x

[45] Yahathugoda T , Wickramasinghe D, Weerasooriya M et al. Lymphoedema and its management in cases of lymphatic filariasis: the current situation in three suburbs of Matara, Sri Lanka, before the introduction of a morbidity-control programme. Ann Trop Med Parasitol. 2005;99(5):501–10. 16004709 10.1179/136485905X46450

[46] Betts H , Martindale S, Chiphwanya J et al. Significant improvement in quality of life following surgery for hydrocoele caused by lymphatic filariasis in Malawi: a prospective cohort study. PLoS Negl Trop Dis. 2020;14(5):e0008314. 32384094 10.1371/journal.pntd.0008314 PMC7239494

[47] Chevarley FM , Thierry JM, Gill CJ et al. Health, preventive health care, and health care access among women with disabilities in the 1994–1995 National Health Interview Survey, Supplement on Disability. Womens Health Issues. 2006;16(6):297–312. 17188213 10.1016/j.whi.2006.10.002

[48] Turner RJ , Beiser M. Major depression and depressive symptomatology among the physically disabled: assessing the role of chronic stress. J Nerv Ment Dis. 1990;178(6):343–50. 2140853 10.1097/00005053-199006000-00001

[49] Kim I . Healthy People 2010 Midcourse Review: progress towards objectives on nutrition and overweight. FASEB J. 2006;20(4):A562.

[50] Bartlett J , Deribe K, Tamiru A et al. Depression and disability in people with podoconiosis: a comparative cross-sectional study in rural Northern Ethiopia. Int Health. 2015;8(2):124–31. 26113669 10.1093/inthealth/ihv037 PMC4604655

[51] Jullien P , Somé JdA, Brantus P et al. Efficacy of home-based lymphoedema management in reducing acute attacks in subjects with lymphatic filariasis in Burkina Faso. Acta Trop. 2011;120(Suppl 1):S55–61. 21470557 10.1016/j.actatropica.2011.03.007

[52] Dreyer G , Medeiros Z, Netto MJ et al. Acute attacks in the extremities of persons living in an area endemic for bancroftian filariasis: differentiation of two syndromes. Trans R Soc Trop Med Hyg. 1999;93(4):413–7. 10674092 10.1016/s0035-9203(99)90140-2

[53] Phillips C , Samuel A, Tiruneh G et al. The impact of acute adenolymphangitis in podoconiosis on caregivers: a case study in Wayu Tuka woreda, Oromia, Western Ethiopia. ‘If she was healthy, I would be free’. PLoS Negl Trop Dis. 2019;13(7):e0007487. 31283763 10.1371/journal.pntd.0007487 PMC6638979

[54] Martindale S , Mackenzie C, Mkwanda S et al. “Unseen” caregivers: the disproportionate gender balance and role of females in the home-based care of lymphatic filariasis patients in Malawi. Front Womens Health. 2017;2(2):1–3.

[55] Mwingira U , Chikawe M, Mandara WL et al. Lymphatic filariasis patient identification in a large urban area of Tanzania: An application of a community-led mHealth system. PLoS Negl Trop Dis. 2017;11(7):e0005748–. 28708825 10.1371/journal.pntd.0005748 PMC5529014

[56] Kebede B , Martindale S, Mengistu B et al. Integrated morbidity mapping of lymphatic filariasis and podoconiosis cases in 20 co-endemic districts of Ethiopia. PLoS Negl Trop Dis. 2018;12(7):e0006491. 29965963 10.1371/journal.pntd.0006491 PMC6044548

[57] Karim MJ , Haq R, Mableson HE et al. Developing the first national database and map of lymphatic filariasis clinical cases in Bangladesh: Another step closer to the elimination goals. PLoS Negl Trop Dis. 2019;13(7):e0007542. 31306409 10.1371/journal.pntd.0007542 PMC6658114

[58] Lotfaliany M , Bowe SJ, Kowal P et al. Depression and chronic diseases: co-occurrence and communality of risk factors. J Affect Disord. 2018;241:461–8. 30149333 10.1016/j.jad.2018.08.011

[59] Dellar R , Ali O, Kinfe M et al. Effect of a community-based holistic care package on physical and psychosocial outcomes in people with lower limb disorder caused by lymphatic filariasis, podoconiosis, and leprosy in Ethiopia: results from the EnDPoINT pilot cohort study. Am J Trop Med Hyg. 2022;107(3):624–31. 35895351 10.4269/ajtmh.21-1180 PMC9490655

[60] Moussavi S , Chatterji S, Verdes E et al. Depression, chronic diseases, and decrements in health: results from the World Health Surveys. Lancet. 2007;370(9590):851–8. 17826170 10.1016/S0140-6736(07)61415-9

[61] World Health Organization . Global report on neglected tropical diseases 2023. Geneva: World Health Organization; 2023.

[62] Harrington A . The placebo effect: an interdisciplinary exploration. Cambridge, MA: Harvard University Press; 1999.

[63] Hamill LC , Haslam D, Abrahamsson S et al. People are neglected, not diseases: the relationship between disability and neglected tropical diseases. Trans R Soc Trop Med Hyg. 2019;113(12):829–34. 31111941 10.1093/trstmh/trz036 PMC6903785

[64] Koschorke M , Al-Haboubi Y, Tseng P-C, Semrau M, Eaton J. Mental health, stigma, and neglected tropical diseases: A review and systematic mapping of the evidence. Frontiers in Tropical Diseases. 2022;3. 10.3389/fitd.2022.808955

[65] Semrau M , Ali O, Deribe K et al. EnDPoINT: protocol for an implementation research study to integrate a holistic package of physical health, mental health and psychosocial care for podoconiosis, lymphatic filariasis and leprosy into routine health services in Ethiopia. BMJ Open. 2020;10(10):e037675. 10.1136/bmjopen-2020-037675 PMC7566734 33060082

[66] Mieras LF , Anand S, van Brakel WH et al. Neglected Tropical Diseases, Cross-Cutting Issues Workshop, 4–6 February 2015, Utrecht, the Netherlands: meeting report. Int Health. 2016;8(Suppl 1):i7–11. 26940311 10.1093/inthealth/ihw001

[67] Jones A , Woods M, Malhotra K. Critical examination of skin care self-management in lymphoedema. Br J Community Nurs. 2019;24(Suppl 10):S6–10. 10.12968/bjcn.2019.24.Sup10.S6 31604041

