# Supplementary figures and images for: Mental health conditions in people affected by filarial lymphoedema in Malawi: prevalence, associated risk factors and the impact of an enhanced self-care intervention
Source: Int Health. 2023 Dec 20;15(Suppl 3):iii14–27. doi: 10.1093/inthealth/ihad064 (PMC10732670; doi:10.1093/inthealth/ihad064)

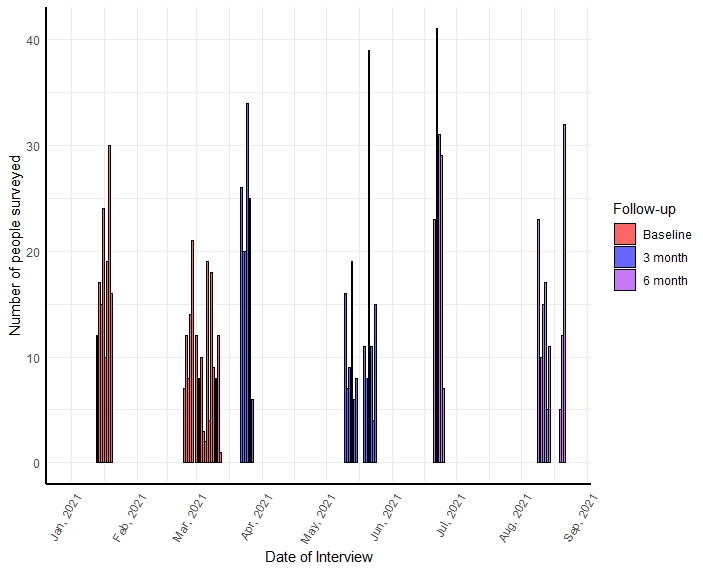

Supplement: ihad064_Supplemental_Figure [file ihad064_supplemental_figure.jpeg]
